# Revolutionizing acute myeloid leukemia treatment: a systematic review of immune-based therapies

**DOI:** 10.1007/s12672-025-01797-9

**Published:** 2025-01-26

**Authors:** Ugochi Ebinama, Binsah George

**Affiliations:** 1 Department of Internal Medicine, The University of Texas Health Sciences Center at Houston, McGovern Medical School, Houston, TX USA; 2 Division of Hematology/Oncology, The University of Texas Health Sciences Center at Houston, McGovern Medical School, 6431 Fannin Street, MSB 5.216, Houston, TX 77030 USA

**Keywords:** Acute myeloid leukemia, Monoclonal antibodies, Unconjugated antibody, Toxin-conjugated antibody

## Abstract

The established protocol for the management of acute myeloid leukemia (AML) has traditionally involved the administration of induction chemotherapy, followed by consolidation chemotherapy, and subsequent allogeneic stem cell transplantation for eligible patients. However, the prognosis for individuals with relapsed and refractory AML remains unfavorable. In response to the necessity for more efficacious therapeutic modalities, targeted immunotherapy has emerged as a promising advancement in AML treatment. This comprehensive review article specifically examines classical unconjugated and toxin-conjugated monoclonal antibodies, which are currently in the preclinical phase or undergoing evaluation in clinical trials. The review delves into the proposed mechanisms through which these monoclonal antibodies elicit anti-tumor activity and identifies the challenges associated with designing targeted immunotherapy. The review focuses on targeting specific antigens in AML, including FLT3/CD125, CLL-1, CD33, CD38, CD47, CD70, and CD123.

## Introduction

Approximately 15% to 20% of leukemia cases in the Western world are associated with Acute myeloid leukemia (AML) [1–4]. Advances in understanding the mechanism and pathophysiology of AML have facilitated the rapid development of targeted treatments for specific AML subsets [4–7]. Traditionally, standard AML treatment involved induction chemotherapy using the “3 + 7” regimen (comprising 3 days of an anthracycline and 7 days of cytarabine), followed by consolidation chemotherapy. The method has resulted in a 30–35% survival rate for patients younger than 60 and 10–15% for those 60 or older over approximately 5 years. Individuals with relapsed AML typically undergo allogeneic stem cell transplantation, but the survival benefits of this procedure are limited [3, 8].

Since 2017, the U.S. Food and Drug Administration has approved nine treatments for various subsets of AML. However, despite these advancements, the 5-year survival rate for relapsed/refractory (R/R) AML remains below 40% [4, 6, 9].

A subset of leukemic cells in AML comprises leukemic stem cells (LSC), which can self-renew and repopulate leukemia cells while demonstrating resistance to chemotherapy. This unique capacity is considered the primary cause of relapsed or recurrent AML. Most LSC share similar cell-cycle properties with normal hematopoietic stem cells (HSC), such as cell-cycle quiescence, enabling them to withstand the effects of chemotherapy and contribute to disease relapse [1, 4, 10]. Ongoing efforts aim to develop targeted immunotherapeutic agents to address this clinically significant pathway and overcome immune tolerance. However, the heterogeneity of AML blasts presents challenges in identifying an ideal target antigen that is predominantly expressed on LSC rather than HSC and in ensuring effective internalization of the antibody–drug conjugate. While LSC exhibits diverse phenotypes, studies have identified the CD34+/CD38− phenotype as the most relevant, also observed in HSC. To advance novel therapies, particularly for relapsed leukemia, researchers are concentrating on identifying and targeting cell surface markers to differentiate LSC from HSC. Progress in AML immunotherapy is rapidly advancing, with researchers exploring various antigen targets over the years, including FLT3/CD125, CLL-1, CD33, CD38, CD47, CD70, and others [3, 11, 12].

The recent advent of molecularly targeted therapies such as IDH1/IDH2/FLT3 inhibitors and the incorporation of the BCL-2 inhibitor Venetoclax in newly diagnosed AML patients have resulted in enhancements in progression-free survival (PFS) and overall survival (OS). Nevertheless, durable remission is not universal among patients, and the potential for relapse persists. Prospective strategies to enhance AML outcomes and long-term survival may entail targeting multiple pathways through immunotherapy, chemotherapy, and molecular therapy synergistically or sequentially. This review offers a comprehensive overview of the advancements in developing immunotherapeutic agents for AML.

## Mechanisms of monoclonal antibodies

The German scientist Paul Ehrlich initially conceived delivering a drug directly to its target in 1913 [Br Med J, 1913. 2(2746): p. 353–9]. At that time, chemotherapy and radiation were the predominant forms of cancer treatment. However, the necessity for targeted therapeutics arose due to their inability to differentiate between healthy and tumor cells. Monoclonal antibodies (Mab) have progressed from murine technology to chimeric, humanized, and ultimately fully humanized forms to reduce immunogenicity. The first monoclonal antibody drug, OKT3 (muromonab CD3), was authorized in 1986 to prevent renal transplant rejection. Subsequently, the chimeric Mab Rituxan was approved in the late 1990s for low-grade B lymphoma, and Humira, a fully humanized Mab, was also approved. These authorizations paved the way for over 100 antibody therapeutics that have been marketed and FDA-approved by March 2017 [Curr Clin Pharmacol, 2018. 13(2): p. 85–99. Lancet, 2019. 394(10200): p. 793–804].

In the 1960s, Mathe introduced the concept of allogeneic hematopoietic stem cell transplant (HSCT), which paved the way for adoptive immunotherapy for AML. This treatment involved the graft-versus-leukemia effect, where immune cells from donor grafts could eliminate leukemia cells [4].

The development of antibody-based therapeutics includes utilizing both unconjugated and toxin-conjugated antibodies. Monoclonal antibodies (mAbs) comprise two matching heavy chains and identical light chains linked by disulfide bonds, creating the recognizable “Y” shaped structure [4, 10].

The variable regions (VH and VL) of the heavy and light chains contribute to the antigen-binding affinity and specificity of mAbs. Additionally, the fragment crystallized (Fc) portion of mAbs contains regions that enable the binding of innate immune cells to Fcγ receptors or complement [4, 13].

Conventional monoclonal antibodies primarily function in cancer therapeutics through natural killer (NK) cell antibody-dependent cell-mediated cytotoxicity (ADCC) [4, 14, 15]. Human NK cells express Fcγ receptors (FcγR), namely FcγRIIIA (CD16a) and FcγRIIC/CD32c, which bind to the Fc segment of monoclonal antibodies. Upon interaction with an antibody-coated target, an immunologic synapse leads to the domination of activation signals over inhibitory signals, ultimately resulting in a cytolytic response [4, 14].

**Fig. 1:**
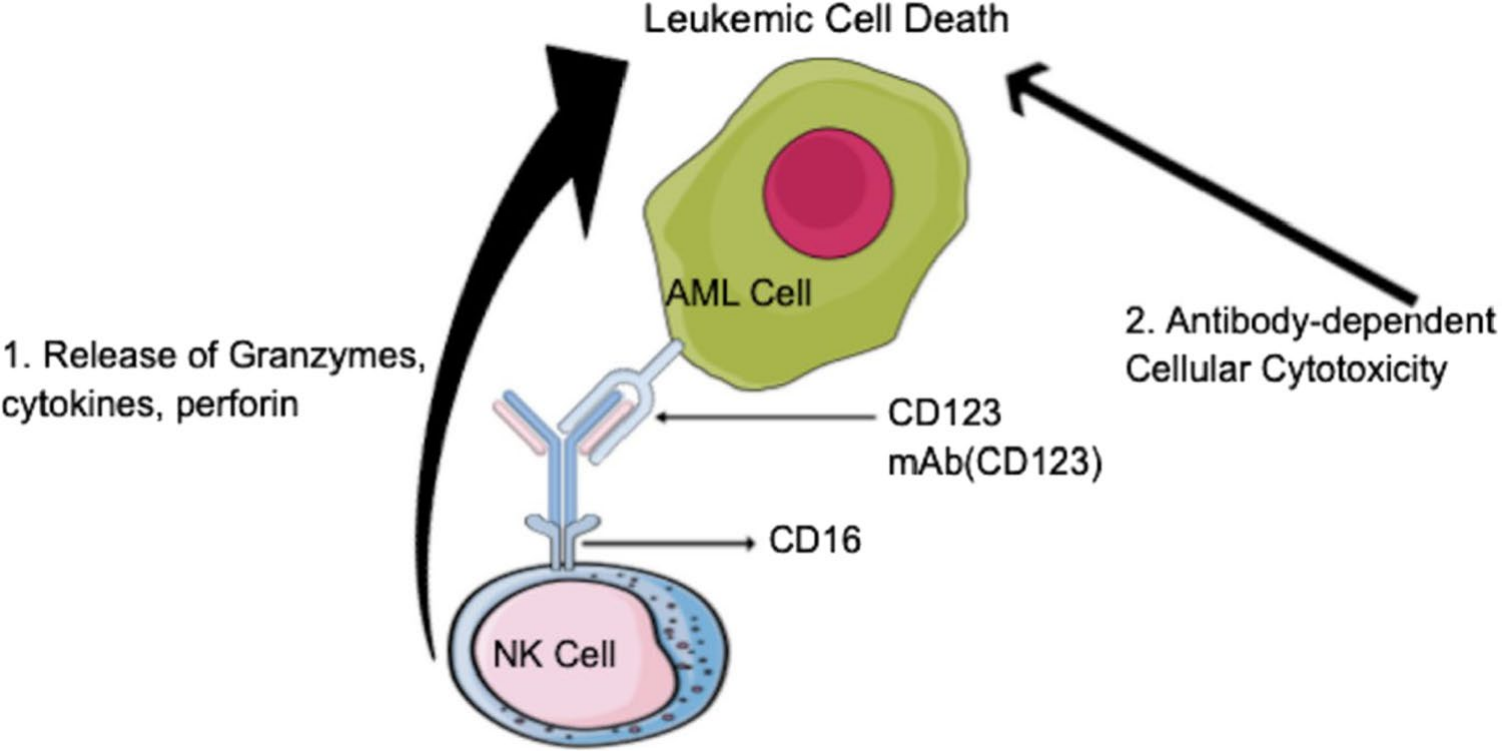
(1) Antibody-dependent NK-mediated tumor lysis occurs by exocytosis of cytotoxic granules containing perforins and granzymes onto the target cell in addition to stimulating cytokine release. (2) A native IgG via antibody-dependent cell-mediated cytotoxicity (ADCC); in this case, anti-CD123 monoclonal antibody [1–4]

Various pathways exist for antibody-dependent NK-mediated destruction of tumors. One such pathway involves the release of cytotoxic granules containing perforins and granzymes onto the target cell (Fig. 1). Perforins can form pores in the target cell membrane, allowing granzymes and serine proteases to enter and cause DNA damage, ultimately leading to apoptosis of the target cell. Granzyme B activates procaspase three, releasing proapoptotic factors that cause DNA damage through caspase-dependent pathways [4].

Research has demonstrated that a specific subset of NK cells, CD56dimCD16+ NK cells, play a crucial role in the cytotoxicity of tumor cells mediated by monoclonal antibodies. Upon activation, these NK cells induce the release of IFNγ, activating neighboring antigen-presenting immune cells, thus stimulating the adaptive immune system [4, 15]. Antibody-dependent phagocytosis by neutrophils and macrophages represents another mechanism for initiating an anti-tumor immune response. These immune cells express all classes of FcγRs. Macrophages and neutrophils phagocytose leukemia cells coated with antibodies, facilitating antigen presentation and activating the adaptive immune system to eliminate tumor cells. Complement-dependent cytotoxicity (CDC) through the classical pathway is the process by which monoclonal antibodies induce cytotoxicity against cancer cells [4]. The toxin-conjugated antibody involves the covalent attachment of a targeted antibody to a cytotoxic drug. Developing an antibody–drug conjugate (ADC) integrates a monoclonal antibody, a cytotoxic agent, and a conjugate linker. The monoclonal antibodies are engineered to bind the antigen target with high affinity and can be humanized, chimeric, or human. Whether cleavable or non-cleavable, the linker is designed to ensure durable binding, thereby preventing premature de-conjugation. ADCs rely on lysosomal degradation to create pores to deliver the cytotoxic drug into the target tumor cells [4, 16, 17]. This review will encompass completed and ongoing clinical trials involving unconjugated mAbs and toxin-conjugated antibodies in AML treatment. Additionally, the review will address other immunotherapy options for AML treatment (Table 1).

**Table 1:** Clinical trials of monoclonal antibodies in AML

NCI clinical trial	Phase	Target antigen	Agents	Target population	Estimated start and end dates	Status
NCT02472145 [4, 18]	II and III	CD123	Decitabine in combination with Talacotuzumab vs. Decitabine alone	365 years of age with de novo or secondary AML not eligible for intensive chemotherapy	August 2015, January 2018	Completed
NCT04778397 [19]	III	CD47	Magrolimab in combination of Azacitidine vs. Venetoclax in combination with Azacitidine or intensive chemotherapy	318 years of age with treatment naïve TP53 AML	March 2021, August 2025	On hold
NCT04755244 [20]	I and II	CD47	ALX148 in combination with Venetoclax and Azacitidine	318 years of age with R/R AML or treatment naïve AML patients, ineligible for intensive chemotherapy	May 2021, December 2023	Completed
NCT04150887 [20]	Ib	CD70	Cusatuzumab in combination with Venetoclax and Azacitidine	318 years of age treatment naïve AML patients, ineligible for intensive chemotherapy	December 2019, June 2023	Completed
NCT04023526 [21]	II	CD70	Cusatuzumab in combination with Azacitidine	318 years of age treatment naïve AML patients, ineligible for intensive chemotherapy	July 2019, August 2023	Active
NCT02632721 [22]	I and II	CD33	BI 836858 in combination with Decitabine	318 years of age treatment with R/R AML	June 2016, January 2023	Active
NCT03067571 [23, 24]	II	CD38	Daratumumab monotherapy	318 years of age treatment with R/R AML or High-Risk MDS	October 2017, November 2022	Completed
NCT00887926 [13, 25]	I	FLT3	IMC-EB10 monotherapy	318 years of age treatment with R/R AML	June 2019, August 2010	Terminated
NCT02785900 [26]	III	CD33	Vadastuximab Talirine combined with HMA vs. Placebo with combination HMA	318 years of age with de novo or secondary AML	May 2016, October 2017	Terminated
NCT03298516 [27]	I	CLL-1	DCLL9718S monotherapy in vs. DCLL9718S in combination with Azacitidine	318 years of age with R/R AML or treatment naïve AML patients, ineligible for intensive chemotherapy	November 2017, July 2019	Completed
NCT03531918 [28]	I and II	CD33	Gemtuzumab Ozogamicin in combination with G-CSF, Cladribine Cytarabine, and Mitoxantone	318 years of age with diagnosis of untreated “high-grade” myeloid neoplasm (≥ 10% blasts in blood or bone marrow) or acute myeloid leukemia (AML) other than acute promyelocytic leukemia (APL) with t(15;17)(q22;q12)	September 2018, July 2025	Active
NCT03737955 [28, 29]	II	CD33	Gemtuzumab Ozogamicin monotherapy	32 years of age with AML in CR and have MRD post-induction chemotherapy	November 2018, June 2024	Recruiting

## Unconjugated monoclonal antibodies for AML treatment

### Talacotuzumab CSL362

Talacotuzumab, a second-gen monoclonal antibody that focuses on CD123, has shown effectiveness in treating CD123, an alpha subunit of the interleukin-3 receptor (IL-3R) that is expressed excessively in hematologic malignancies, such as AML [4, 18]. Studies suggest that leukemic stem cells express CD123 at higher rates in the CD34+/CD38− region and at lower rates in HSCs. The Fc region of the monoclonal antibody binds to CD16a of NK cells, and the antibody’s variable region shows an affinity to bind CD123 [4].

A phase I clinical trial involving CD123+ patients in the first or second CR during the consolidation phase demonstrated the general tolerability of Talacotuzumab. The interim report reported only three severe adverse events among 25 patients.

Furthermore, 10 patients maintained complete remission for over 6 months, with three out of six MRD-positive patients transitioning to MRD-negative status [4, 13, 18].

However, after a phase II clinical trial (NCT02472145) evaluated Talacotuzumab, further advancement was halted because it failed to demonstrate efficacy and improved overall survival outcomes.

These findings suggest that CD123 holds promise as a target for developing monoclonal antibodies to induce ADCC, particularly in patients at risk for relapsed or refractory AML [4, 18].

### ALX148 (Evorpacept)

The monoclonal molecule ALX148 has recently been developed to target CD47. This is achieved by fusing a modified SIRPα domain 1 (D1) with a humanized IgG1 Fc that lacks activity. This fusion enhances macrophages’ capacity to engulf tumor cells [18]. Studies on murine tumor xenograft models have demonstrated that ALX148 can amplify the antitumor effects of specific monoclonal antibodies. Furthermore, the blockade of CD47 with ALX148 elicits diverse immune responses within the adaptive and innate immune systems [18, 20].

ASPEN-05 clinical trial (document number 1) constitutes a phase 1/2 clinical trial designed to appraise the effectiveness of ALX148 when administered alongside venetoclax (VEN) plus azacitidine (AZA) in patients diagnosed with AML. The trial's Phase 1a segment encompassed patients with R/R AML or newly diagnosed AML with high-risk cytogenetics who were unsuitable for induction chemotherapy. These patients were stratified into groups receiving increasing doses of ALX148 (20 mg/kg Q2W, 30 mg/kg Q2W, and 60 mg/kg Q4W) concomitantly with VEN (400 mg PO daily for up to 28 days) and AZA (75 mg/m^2^ IV/SC daily for 7 days) in 28-day cycles. The primary objective was to monitor any dose-limiting toxicities (DLTs), while the secondary goal was to evaluate the anti-leukemic activity of ALX148 in combination with VEN and AZA [18, 20, 30].

Preliminary results indicate that patients exhibited good tolerance to Evaporcept in conjunction with standard VEN and AZA, and no maximum-tolerated dose (MTD) was identified. The highest administered dose of Evaporcept, set at 60 mg/kg, led to a reversible grade 3 cytokine release syndrome (CRS). Additionally, CD47 activity was observed in both peripheral blood and bone marrow, demonstrating anti-leukemic effects across all Evaporcept doses. These findings suggest the potential effectiveness of ALX148 in treating myeloid malignancies [20].

### Cusatuzumab

Cusatuzumab, a monoclonal antibody primed for antibody-dependent cell-mediated cytotoxicity (ADCC), targets the CD70–CD27 signaling expressed by AML stem and progenitor cells. This signaling activates stem cell gene expression, thus promoting balanced cell division and proliferation. Studies on AML xenografts demonstrated that inhibiting the CD70/CD27 interaction with monoclonal antibodies led to asymmetric cell division and proliferation of AML LSCs and AML blasts, inhibited cell growth, and extended survival [18, 31, 32].

Furthermore, normal HSCs do not express CD70 or CD27, making a monoclonal antibody targeting the CD70/CD27 signaling a promising therapeutic option for AML. A phase I/II clinical trial (NCT0303612) assessed the efficacy of Cusatuzumab in combination with azacitidine for treating treatment-naïve AML patients. During the study, twelve patients received 2 weeks of cusatuzumab, followed by the addition of azacitidine. Out of the 12 patients enrolled, 8 achieved CR, 2 CRi, and two partial responses; four patients were MRD negative by flow cytometry. Cusatuzumab significantly reduced LSCs, indicating its enduring potential in AML treatment [18, 21, 33].

CULMINATE (NCT04023526) is a multicenter phase I/II clinical trial aimed at evaluating the efficacy of Cusatuzumab combined with azacitidine (AZA) for the treatment of newly diagnosed AML patients ineligible for intensive chemotherapy. Cusatuzumab was administered at various doses (1, 3, 10, or 20 mg/kg) for 14 days before commencing combination therapy with AZA.

During the phase I dose escalation, Cusatuzumab was co-administered with AZA (75 mg/m^2^) from days 1 through 7 of a 28-day cycle. The results demonstrated that 50% of the treated patients achieved an objective response (CR or CRi). It was established that the recommended efficacious dose was 10 mg/kg, and this combination therapy exhibited general tolerability without any observed dose-dependent toxicities. The median response duration was 4.5 months, with an overall survival (OS) of 11.5 months [34].

ELEVATE (NCT04150887) is an active phase 1b clinical trial assessing Cusatuzumab combined with venetoclax with or without azacitidine [18, 35].

### Lintuzumab

Lintuzumab is a monoclonal antibody that targets CD33, a marker found on myeloid progenitors and is expressed in 20–25% of leukemic blasts [36, 37]. Several clinical trials, including NCT00002609, NCT00002800, NCT00006084, NCT000016159, NCT0000283114, NCT00502112, NCT00528333, and NCT00997243, have evaluated the effectiveness of Lintuzumab in combination with induction and maintenance chemotherapy for patients with relapsed or refractory acute myeloid leukemia (AML). However, these phase I, II, and III clinical trials did not demonstrate any overall survival benefit, leading to the discontinuation of further development of unconjugated Lintuzumab [36].

Rosenblat et al. conducted the first human trial using Lintuzumab conjugated to the actinium isotope ^225^Ac to enhance its antileukemic activity. When infused, this isotope decays into four alpha emissions; alpha particles have been shown to effectively kill tumor cells in vitro with minimal off-target cytotoxic side effects. Eighteen patients with relapsed or refractory AML were enrolled in the study and received an infusion of ^225^Ac-lintuzumab at five doses ranging from 18.5 to 148 kBq/kg, with a maximum tolerated dose (MTD) determined to be 111 kBq/kg. Although the patients did not achieve remission, antileukemic effects were observed 1 month post-treatment at doses greater than 37 kBq/kg: a decrease in peripheral blasts was noted in 63% of the patients, and more than a 10% reduction in bone marrow blasts was seen in 67% of the patients. One patient achieved a leukemia-free state and progressed to allogeneic hematopoietic cell transplantation (alloHCT) but unfortunately succumbed to complications from the transplant.

The significant toxicities observed included myelosuppression and hepatotoxicity. One patient experienced prolonged myelosuppression lasting more than 35 days at the 148 kBq/kg dose, while two patients died at the doses of 111 kBq/kg and 148 kBq/kg, respectively. This study demonstrated the tolerability and feasibility of using the targeted alpha-particle generator 225Ac-lintuzumab. Future trials could explore the potential of combining ^225^Ac-lintuzumab with standard chemotherapy to promote durable responses in relapsed or refractory AML [36].

Moreover, the monoclonal antibody BI-836858 does not undergo conjugation and has an improved Fc region that boosts NK-mediated ADCC compared to the original Fc region. A phase I/II clinical trial (NCT02632721) is underway to establish the maximum tolerated dose and examine the safety and efficacy of BI 836858 combined with Decitabine in individuals with relapsed or refractory AML [4, 22].

### Daratumumab (Darzalex), Isatuximab

Daratumumab, an IgG1 kappa monoclonal antibody, targets the CD38 protein, expressed in myeloid, lymphoid, red cells, and platelets, with heightened expression in plasma cells [18, 38]. Both Daratumumab and Isatuximab have effectively treated refractory multiple myeloma [38]. In vitro studies have revealed that Darzalex induces apoptosis in primary AML targets through cytotoxic mechanisms such as ADCC and CDC. Additionally, studies using primary AML xenografts have shown that Daratumumab reduces the burden of leukemia in the peripheral blood and spleen, except the bone marrow, through the ADCC, CDC, and ADCP mechanisms [4, 18, 39]—a completed phase II clinical trial conducted by M.D. Anderson Cancer Center, with the identifier NCT03067571, evaluated the efficacy and safety of daratumumab in patients with relapsed/refractory and high-risk MDS [4, 23, 24]. The results of this study are not currently available.

## Toxin-conjugated monoclonal antibodies for AML treatment

### Gemtuzumab Ozogamicin (Mylotarg)

Gemtuzumab Ozogamicin (GO) is a humanized anti-CD33 antibody conjugated with calicheamicin, and the initial ADC was authorized as a therapeutic agent for hematologic malignancies [4, 18, 29]. CD33 is expressed on blasts and LSCs at a rate of 85–90% [1, 12, 40]. In 2000, the FDA sanctioned GO monotherapy (9 mg/m^2^ repeated in 14 days for two doses) following a phase III clinical trial exhibiting a 30% CR in patients aged over 60 years with relapsed AML. However, toxicities were observed at this dose [18, 41–43].

2010, the drug GO was recalled following the phase III SWOG post-approval trial results. This trial, conducted across multiple centers, compared the administration of GO at a dosage of 6 mg/m^2^ on day 4 of daunorubicin and cytarabine induction chemotherapy. The trial did not demonstrate improvements in complete remission (CR), relapse-free survival (RFS), and overall survival (OS) in patients aged 65 or younger. Consequently, the decision was made to voluntarily withdraw the agent from the market [18, 42, 44].

Subsequent trials assessed lower dosages of GO in combination with chemotherapy, focusing on finding the most efficacious dose with minimal severe toxicities such as veno-occlusive disease (VOD) [4, 18].

In the MRC AML-15 study, which involved 1113 previously untreated AML patients, the comparison of induction/consolidation chemotherapy with or without 3 mg/m^2^ GO revealed no disparities in overall survival, relapse-free survival, overall response rate, toxicities, or 30-day all-cause mortality between the cohort that received GO combined with chemotherapy and the one that received chemotherapy alone. Nevertheless, this investigation demonstrated enhanced overall survival for the group receiving the GO combination with chemotherapy among young patients with favorable risk cytogenetics (Core-binding factor (CBF-AML), a modest improvement in intermediate-risk AML, and no advantage in high-risk cytogenetics. These observations were also noted in several earlier clinical trials [18, 45]. Recent clinical trials have similarly exhibited the survival advantages of combining low-dose GO with intensive chemotherapy in older AML patients [4, 18, 29].

The MRC AML-16 trial demonstrated a meaningful improvement in 3-year cumulative relapse (68% vs. 75%) and overall survival (25 vs. 20%) in older patients who received combination chemotherapy and 3 mg/m^2^ GO compared to those who received chemotherapy alone [4, 18, 29]. ALFA-0701, a phase III clinical trial, involved 271 older patients with treatment-naïve de novo AML (aged 50 to 70 years) randomly assigned to receive GO combined with chemotherapy or chemotherapy alone. GO 3 mg/m^2^ was administered on days 1, 4, and 7 during induction and on day 1 of the two consolidation cycles [18, 46, 47].

Older patients with favorable and intermediate cytogenetics who received GO combined with chemotherapy had improved median event-free survival and overall survival compared to the chemotherapy-alone group. However, there were no differences in early mortality [18, 47].

A few studies have shown the improved outcomes of GO monotherapy in older AML patients not eligible for intensive chemotherapy. AML-19 is the first phase III clinical trial to show significantly improved OS with a single-agent GO in this patient population. In a subsequent phase II clinical trial, MyloFrance-1, the second remission was achieved in 33% of relapsed AML patients with GO monotherapy [4, 18, 48]. The outcomes of these clinical trials led to FDA approval in 2017 of GO as monotherapy or combined with intensive chemotherapy for CD33-positive relapsed/refractory AML [4, 18]. An ongoing phase I/II clinical trial assessing the efficacy of single or fractionated Gemtuzumab Ozogamicin in combination with G-CSF, Cladribine, Cytarabine, and Mitoxantone (GCLAM) in treatment naïve AML patients or high-grade myeloid neoplasms (≥ 10% blasts in blood or bone marrow) in patients greater than 18 years of age. The study excluded patients with Acute Promyelocytic Leukemia, t(15;17) (q22;q12). It aims to assess the maximum tolerated dose (MTD) of GO when combined with GCLAM, event-free survival (EFS), measurable residual disease (MRD), and remission rates (i.e., CR) [28]. A phase II clinical trial (NCT03737955) is ongoing to assess the efficacy of GO monotherapy in treating measurable residual disease in AML.

### FLT3 antibody-drugs conjugate

Roughly 30% of patients diagnosed with AML exhibit FMS-like tyrosine kinase 3 (FLT3) gene mutations. In AML blast cells, FLT3 is frequently observed to be overexpressed, with the most prevalent mutation being internal tandem duplication (FLT3-ITD) [4, 49]. This specific mutation is associated with an unfavorable prognosis and is correlated with higher rates of relapse or refractory disease, as well as poorer overall outcomes. The accrual of new mutations in the FLT3 gene over time is believed to contribute to relapse in AML patients with FLT3-ITD, in addition to selecting subclones resistant to chemotherapy. Consequently, it is recommended that patients undergo testing for FLT3-ITD both at the initial diagnosis of AML and during relapse. Moreover, approximately 5% of AML patients harbor FLT3-tyrosine kinase domain (TKD) mutations, activating kinase and uncontrolled cellular proliferation. The prognostic significance of FLT3-TKD mutations remains to be determined [18, 49, 50].

The current standard induction therapy for newly diagnosed patients with FLT3 mutations in AML involves a combination of 7 + 3 chemotherapy and Midostaurin [51]. Gilteritinib monotherapy is the FDA-approved first-line therapy, followed by hematopoietic stem cell transplantation (HSCT) and maintenance therapy for those achieving complete remission (CR) for R/R AML with FLT3 mutations. Gilteritinib inhibits the FLT3 tyrosine kinase inhibitors and downstream pathways such as STAT5. The major adverse event observed with Gilteritinib was myelosuppression.

A phase I clinical trial (identified as NCT00887926) assessed the maximum tolerated dose (MTD) and pharmacokinetic profile of single-agent IMC-EB10, an anti-FLT3 monoclonal antibody, in patients with relapsed/refractory AML. Preclinical models demonstrated the anti-leukemic activity of IMC-EB10 using antibody cell-mediated cytotoxicity (ADCC). While the study revealed a favorable safety profile for the medication, it lacked clinical efficacy, leading to the termination of the trial [13, 25].

### Vadastuximab Talirine (SGN33A)

Vadastuximab talirine (SGN33A) is an antibody–drug conjugate undergoing assessment to treat relapsed or refractory acute myeloid leukemia (AML) [4]. CD33, the drug's target, is known to be overexpressed in AML patients. This drug consists of a pyrrolobenzodiazepine dimer linked to a monoclonal antibody that targets explicitly CD33. In a phase I clinical trial evaluating SGN33A as monotherapy in patients with relapsed or refractory AML, the 30-day mortality rate was 8%, with a complete remission or incomplete hematologic recovery (CRi) rate of 28%. Approximately 50% of the responding patients exhibited minimal residual disease (MRD). The primary adverse event observed was myelosuppression [4, 18, 52]. Although these results were promising, the outcomes of the phase III clinical trial, known as the CASCADE trial, led to the discontinuation of further development of vadastuximab taurine as monotherapy in newly diagnosed older AML patients. The CASCADE trial assessed the efficacy of SGN33A with or without hypomethylating agents (azacitidine or decitabine) and was terminated due to significant myelosuppressive toxicity in the SGN33A treatment arm [18, 26, 53].

### Anti–CLL-1 ADC

The C-type lectin-like molecule-1 (CLL-1) is a transmembrane glycoprotein overexpressed in AML blasts and LSCs. Its absence in normal HSCs makes it an ideal therapeutic target [27]. A phase I dose escalation study of DCLL9718S involved an ADC composed of a humanized monoclonal anti-CLL-1 antibody linked to pyrrolobenzodiazepine (PBD) dimer drugs through disulfide bonds. This study enrolled 18 patients with R/R AML distributed across five dose-escalating cohorts with 10–160 μg/kg doses. None of the 18 patients achieved complete remission (CR) or partial remission (PR), irrespective of the dose. The study was terminated during dose escalation due to severe hepatotoxicity and the observed lack of antileukemic activity [18, 27].

### Tagraxofusp (SL-401)

The alpha chain of the interleukin-3 receptor (CD123) is expressed in various hematologic neoplasms, including AML [13, 54]. Tagraxofusp (TAG) is the first monoclonal antibody targeting anti-CD123 to receive FDA approval to treat blastic plasmacytoid dendritic cell neoplasm (BPDCN). AML with expression of CD123 has been associated with minimal residual disease (MRD) following treatment, which is correlated with higher rates of disease relapse. A phase 1b clinical trial (document number 1) investigated a novel treatment approach for high-risk AML involving TAG (12 μg/kg/day for 3 days), AZA for 7 days, and VEN for 21 days. The findings revealed that 39% of the cohort achieved complete remission (CR), 19% achieved full remission with incomplete count recovery (CRi), and 12% achieved a morphologic leukemia-free state (MLFS). Among 13 patients with TP53 mutations, 54% responded favorably (CR/CRi/MLFS). Moreover, 71% of the patients responsive to the TAG/AZA/VEN regimen exhibited no detectable minimal residual disease (MRD). The median overall survival and progression-free survival were 14 and 8.5 months, respectively. These compelling results underscore the potential of TAG combinations as an effective treatment strategy for high-risk AML [55, 56].

### IMGN632 (Pivekimab sunirine)

The novel IMGN632 represents an antibody–drug conjugate (ADC) targeting CD123, comprising an anti-CD123 antibody linked to a DNA mono-alkylating conjugate of the indolinobenzodiazepine pseudodimer (IGN) class of cytotoxic compounds [18, 57]. A phase 1/2 clinical trial investigated dose-escalation and dose-expansion, enrolling adult patients with refractory or relapsed (R/R) acute myeloid leukemia (AML) from the USA, Italy, Spain, and France. The primary objective was to assess the safety and efficacy of IMGN632 in this patient cohort. During the dose-escalation phase (phase 1a), two distinct schedules were employed: Schedule A, administered every 3 weeks on day 1 of a 3-week cycle, and fractionated Schedule B, administered on days 1, 4, and 8 of a 3-week cycle. The dose expansion phase comprised two cohorts receiving 0.045 mg/kg and 0.090 mg/kg, respectively. Analysis indicated no observed maximum tolerated dose (MTD) during the dose-escalating phase, determining the recommended phase 2 dose to be 0.045 mg/kg once every 3 weeks. The primary treatment-related adverse event reported at grade 3 was febrile neutropenia. The overall response rate at the suggested phase 2 dose was 21%, with a complete response (CR) rate of 17%. These findings have led to the initiation of an active phase 1b/2 study of pivekimab sunirine combined with AZA and VEN in CD123 AML patients based on the phase 1a study results [58].

## Immune checkpoint inhibitors (ICI)

ICIs are immune regulation molecules expressed in T-cells designed to disrupt abnormal regulatory circuits between immune cells and cancerous cells. There is compelling evidence that in acute myeloid leukemia (AML), cancer cells evade the immune system by modifying the immune compartment and aberrantly expressing ligands to ICs, directly adapting AML cells.

PD-L1, a programmed cell-death ligand, is an identified ligand in AML that interacts with PD-1 receptors on T-cells, causing T-cell exhaustion (PMID: 30911134). This pathway also supports regulatory T cells (Tregs), further suppressing the function of CD8 T cells (PMID: 33271388). TIM-3, a T-cell immunoglobulin and mucin domain 3, is a well-defined immune checkpoint in both effector T and NK cells. TIM-3 attaches to galectin-9, which is highly expressed on AML blasts, promoting self-renewal through stimulatory β-catenin and NFκB signaling and reducing the release of pro-inflammatory cytokines, ultimately resulting in NK and T-cell dysfunction (Blood et al. 2015;5:330). In recent years, several clinical trials have evaluated the approved ICIs for relapsed or refractory (R/R) AML and AML at high risk of relapse after HSCT or other treatments. However, single-agent ICI therapy has shown limited effectiveness, with survival rates ranging from 10 to 20% (Front Oncol, 2022;12:882531). Combining anti-PD1 and CTLA4 antibodies with antileukemic drugs that have shown immunological effects in other types of tumors is under investigation. For example, hypomethylating agents such as decitabine plus azacitidine are being studied for their ability to stimulate HLA and tumor antigens in numerous solid tumors. Additionally, in colon cancer, the Bcl-2 inhibitor venetoclax is being researched for its ability to enhance effector T cells and the anti-tumor properties of ICI in preclinical models (Cancer Discov, 2021;11:68–79). The combination of nivolumab (an anti-PD1 antibody) with azacitidine resulted in a 56% overall response rate among patients with increased CD3+ T cells in the bone marrow, regardless of whether the patient received treatment for the first time or had been previously treated (Cancer Discovery, 2019; 9:370–83).

However, a recent research study involving older patients found no additional clinical benefit from azacitidine when combined with durvalumab (an anti-PD-L1 antibody) (Clin et al. 2022; 22:S255).

## Macrophage immune checkpoint inhibitors

Macrophages are an essential component of the innate immune system and play a crucial role in the tumor microenvironment at all stages of tumor progression. M1 macrophages suppress tumor growth, while M2 macrophages, including the subtypes M2a, M2b, M2c, and M2d, contribute to tumor angiogenesis, growth, and resistance to therapy. Consequently, various therapeutic strategies have been developed to target M2 macrophages to promote remission and improve patient outcomes. These strategies include reducing the presence of tumor-activating macrophages and monocytes, blocking the recruitment of M2 macrophages and monocytes, or enhancing the phagocytic activity of macrophages.

Additionally, macrophages express several checkpoint receptors implicated in the immunosuppressive tumor microenvironment, such as V-domain Ig suppressor of T cell activation (VISTA), B7-H4, PD-L1, PD-L2, CTLA-4 ligands B7-1 and B7-2, Tim-3, and CD47. Inhibiting these checkpoints can lead to the reprogramming of M2 macrophages into pro-inflammatory M1 macrophages, helping to prevent cancer cells from evading the immune system. However, challenges to the effectiveness of macrophage immune checkpoint inhibitors include resistance based on the type of cancer and the potential for immune-related adverse events (IRAE) in various organs due to the off-target effects of the inhibitors [59].

### Magrolimab

Magrolimab (5F9) is an IgG4 anti-CD47 antibody. Studies have indicated that CD47 is overexpressed in AML LSCs compared to normal HSCs. Previous research involving cohorts has displayed decreased overall survival in AML patients with elevated CD47 levels. CD47 is a transmembrane protein–ligand for signal regulatory alpha (SIRPα). SIRPα is present in macrophages and dendritic cells, and its interaction with CD47 activates a transduction cascade, inhibiting phagocytosis [4, 18, 60–62]. The Phase 1 CAMELLIA clinical trial (NCT02678338) examined magrolimab as a monotherapy in relapsed/refractory AML and high-risk myelodysplastic syndrome (MDS). The trial was halted due to insufficient clinical effectiveness [18, 19]. A phase 1b clinical trial evaluating the combination of magrolimab and azacitidine (AZA) showed clinical efficacy in both treatment naïve high-risk MDS and AML compared to AZA alone. The sample size in this trial (N = 43; AML patients N = 25) includes 28% with TP53 mutation, a poor prognostic marker. In general, treatment with a combination of AZA and magrolimab exhibits tolerability in patients. The most common adverse events were anemia, followed by neutropenia and thrombocytopenia. At the time of data efficacy, 16 patients in AML were evaluable; of those, 50% achieved complete response (CR) or incomplete count recovery (CRi). In the population of AML patients who achieved CR with 5F9 plus AZA, about 37% were negative for measurable residual disease (MRD) through flow cytometry, measured by LSC frequency of CD34+/CD38− with data showing complete elimination of the LSCs [18, 63, 64]. This clinical trial showed a durable response with a combination of AZA plus magrolimab and the potential of CR in TP53-AML subgroups. Azacitidine enhances calreticulin expression on AML cells and macrophage-mediated phagocytosis when combined with magrolimab [18, 64].

## Sabatolimab (MBG453)

Sabatolimab selectively targets TIM3 receptors on immune cells, blasts, and LSCs. Currently, it is being assessed as a component of the STIMULUS (NCT04266301) clinical trial for treating newly diagnosed AML in individuals ineligible for intensive chemotherapy or HSCT and high-risk MDS. The clinical trial showed that combining sabatolimab with hypomethylating agents (HMAs) positively affected safety and tolerance and led to long-lasting clinical benefits. These findings were reported in Blood 2021;138(Suppl, 2021):244 However, the phase III Stimulus trial did not demonstrate efficacy in the primary endpoint of overall survival; therefore, Sabatolimab has been discontinued as a therapeutic candidate for AML.

## Chimeric antigen receptor (CAR) T cell therapy

Chimeric antigen receptor (CAR) T-cell therapies have fundamentally transformed the landscape of hematologic cancers, specifically for conditions such as multiple myeloma, lymphoma, and acute lymphocytic leukemia. Six CAR-T therapeutics have received approval from the United States Food and Drug Administration (FDA), highlighting their significant impact and efficacy in treating these diseases [65].

Among acute leukemias, acute myeloid leukemia (AML) stands out as the most prevalent type affecting adult patients. Below, we describe active clinical trials to evaluate the effectiveness of CAR-T therapies that specifically target unique surface antigens associated with AML. Exploring diverse antigens and optimizing CAR constructs are vital steps in enhancing the precision and outcomes of therapy for AML patients in the future [65, 66].

### CD123 CAR T-cell therapy

Budde et al. were the first to evaluate the efficacy of CD123CAR T cells in a phase I first-in-human clinical trial (NCT02159495) involving six patients with relapsed/refractory acute myeloid leukemia (R/R AML) after undergoing allogeneic hematopoietic cell transplantation (alloHCT) and having a median of four prior therapy lines. All patients in the AML cohort received lymphodepletion chemotherapy before the CAR-T cell infusions. Two patients were infused at dose level 1 (DL1, 50 million cells), while four received infusions at dose level 2 (DL2, 200 million cells).

In the DL1 group, one patient achieved a 2-month leukemia-free state and subsequently underwent a second infusion. After 35 days, flow cytometry showed a reduction in blast cells from 77.9 to 0.9%. In the DL2 group, two patients achieved complete remission (CR) following their initial infusion. They proceeded to a subsequent alloHSCT on day 70 and were minimal residual disease (MRD)-harmful at 161 days post-transplant. The other two patients experienced decreased blast counts, as indicated by flow cytometry. Adverse events reported were reversible (CRS, adenovirus pneumonia, and recurrence of cutaneous GvHD (Table 2). These results suggest that CD123 CAR-T cells may be promising candidates for treating R/R AML [67].

**Table 2:** Treatment-related adverse events for the immunotherapies in AML

Drug	Major toxicities
Talacotuzumab (CSL362)	Myelosuppression
Evaporcept (ALX148)	Urticaria, neutropenia, abdominal disconfort
Cusatuzumab	Neutropenia, Lung infection
Lintuzumab	Myelosuppression
Daratumumab, Isatuximab	Infusion site reaction, edema, neutropenia
Gemtuzumab Ozogamicin	Veno-occlusive disease, Hepatotoxicity
Gilteritinib	Ventricular arrythmia, myelosuppression
Vadastuximab talirine	Venous Thrombosis, myelosuppression
Immune Checkpoint Inhibitors	Immune-related adverse events (gastrointestinal, endocrine, dermatologic, neurotoxicity and pulmonary toxicity)
Chimeric antigen receptor T-cell therapies	CRS, ICANS

An active open-label phase I trial (NCT03766126) currently assesses the efficacy of anti-CD123 CAR-T cells transduced with a lentivirus in patients with relapsed or refractory AML following lymphodepleting chemotherapy. This study is divided into two cohorts: Cohort 1a/1b (utilizing a split-dose approach) and Cohort 2 (which involves a single-dose infusion of anti-CD23 cells; the minimum acceptable dose is 1 × 10^5^ cells/kg). Patients in this study are recommended to undergo autologous HSCT as rescue therapy on Day 28 if they exhibit bone marrow aplasia. Participants will be monitored for 15 months following the initial infusion, with long-term follow-up up to 15 years [67].

### CD7 CAR T-cell therapy

A clinical trial (NCT04538599) conducted by Yongxian et al. evaluated the efficacy of CD7 CAR T-Cell therapy followed by allogeneic hematopoietic stem cell transplantation (alloHSCT) in 17 patients with relapsed/refractory (R/R) CD7-positive leukemia or lymphoma; none of the patients had previously undergone alloHSCT. Of these patients, 16 received donor-derived CD7 CAR T-cells, while one received universal CD7 CAR T-cells. Ten patients subsequently proceeded to haploidentical HSCT. Unfortunately, one patient died due to septic shock and encephalitis.

Among the patients, eight successfully achieved donor engraftment and experienced recovery of allogeneic hematopoiesis, though two of these patients later died from CD7-negative leukemia relapse and infection. Five patients in this group achieved complete remission (CR) and maintained minimal residual disease (MRD)-negative status; however, one eventually progressed to relapsed CD7-negative leukemia. Of the ten patients, one experienced donor engraftment failure with autologous hematopoiesis recovery and maintained MRD-negative CR.

The 1-year overall survival (OS) rate for patients who achieved CR was 68% [68].

### CD33 CAR T-cell therapy

Tamboro et al. conducted a phase I clinical trial (NCT03126864) to assess the effectiveness and tolerability of CD33 CAR T-cells, which incorporate 4-1BB and CD3ζ endo-domains, alongside a truncated version of the human epidermal growth factor receptor (HER1t). The trial involved ten patients with relapsed or refractory acute myeloid leukemia (R/R AML), all of whom had undergone five prior lines of treatment, with three patients having received prior allogeneic hematopoietic stem cell transplantation (alloHSCT). The results of this study indicated a lack of efficacy for CD33 CAR T-cells in R/R AML. Several challenges were encountered during the study, including rapid disease progression, severe lymphopenia, which complicated the collection of adequate T-cells during the apheresis phase, and high blast counts [37].

## Natural killer (NK)-mediated immunotherapy

Natural killer (NK) cells are an essential part of the immune system, recognized for their ability to respond quickly to tumors. They carry out this response through antibody-dependent cellular cytotoxicity (ADCC), where NK cells identify and destroy tumor cells coated with antibodies. Additionally, NK cells react to signals from various cell surface receptors, which fall into two categories: activating receptors and inhibitory receptors.

Activating receptors boost the NK cell’s ability to kill target cells, while inhibitory receptors help regulate this activity, preventing damage to healthy tissues by downregulating NK cell responses. The balance between these activating and inhibitory signals is crucial for maintaining immune homeostasis, allowing the immune system to target tumor cells while effectively protecting normal cells from harm [65, 69].

Bajel et al. conducted an ongoing phase I/II first-in-human clinical trial (NCT05086315) assessing the tolerability and anti-leukemic activity of SAR443579, which is an anti-CD123 NKp46xCD16 natural killer cell engager where NK cells interact with CD123 expressing tumors leading to cytolytic destruction of tumor cells. There were 43 participants enrolled in the study (42 R/R AML and one high-risk MDS) and were administered three 28-day cycle infusions of SAR443579 at varying dose levels once or twice weekly depending on the dose; the patients had a median of 2 prior treatment lines, 36 patients with prior exposure to VEN, and 13 patients with prior alloHSCT. SAR443579 was well tolerated up to a maximum dose of 6000 µg/kg weekly [69].

## Mechanisms of biomarker resistance and challenges of immunotherapy in AML

Acute myeloid leukemia (AML) is a complex blood cancer characterized by various genetic mutations. These mutations can significantly influence the disease’s progression and treatment effectiveness. They may affect crucial cellular pathways and mechanisms, leading to diverse variations in how the disease presents and progresses among patients.

This heterogeneity presents considerable challenges in developing and implementing effective immune-based therapies. The presence of specific genetic alterations can either enhance or reduce the potential effectiveness of these treatments. Therefore, understanding the unique genetic profile of each AML patient is essential for creating personalized therapeutic approaches that maximize treatment responses and improve overall outcomes. Leukemia cells exhibit several mechanisms that contribute to their ability to develop resistance to drug treatments. One critical mechanism is the dormancy of leukemia stem cells (LSCs), which enables them to evade the effects of therapeutic agents that target actively dividing cells. Because many traditional chemotherapies are designed to attack cells during the cell cycle, dormant LSCs can remain unaffected, allowing them to survive and eventually repopulate the cancer [66].

Additionally, LSCs often show overexpression of ATP-binding cassette (ABC) transporters, a family of membrane proteins that expel various drugs from inside the cell. This efflux mechanism effectively reduces the intracellular concentration of chemotherapeutic agents, rendering them less effective and contributing to the overall drug resistance observed in leukemia [66].

Another factor in the resistance phenomenon is the presence of defective apoptotic mechanisms within these LSCs. When the processes that lead to programmed cell death are malfunctioning, LSCs can withstand otherwise lethal doses of drugs. This resilience contributes to drug resistance and the persistence of leukemia in patients [66].

Moreover, epigenetic modifications, such as DNA methylation changes in LSCs, facilitate drug resistance. These alterations can lead to the silencing of genes associated with cell cycle regulation and apoptosis, ultimately contributing to leukemia relapse post-treatment.

On a different note, immune-based therapies are less toxic than conventional chemotherapy regimens but are not without risks. Immune-related adverse events can occur because of these therapies, highlighting the need for careful management and monitoring of patients undergoing such treatments. Striking an optimal balance between treatment efficacy and safety remains a significant challenge in the clinical management of leukemia [70–72].

## Conclusion

Despite advancements in targeted immunotherapy for acute myeloid leukemia (AML), the prognosis for patients with relapsed or refractory AML remains poor. This review explores how unconjugated and conjugated monoclonal antibodies work against leukemia and the various antigen targets currently under investigation in clinical trials.

Most unconjugated monoclonal antibodies have limited efficacy, with Magrolimab being a notable exception. This highlights the challenges of harnessing the immune system effectively, particularly with antibody-dependent cellular cytotoxicity (ADCC). Gemtuzumab Ozogamicin is the only FDA-approved monoclonal antibody for AML, demonstrating a unique mechanism by delivering a cytotoxic agent directly to leukemic cells. However, more therapies are needed for this aggressive disease.

The AML treatment landscape is evolving, with promising avenues such as immune checkpoint inhibitors, radioimmunotherapy, and chimeric antigen receptor T-cell (CAR-T) therapies currently under investigation. Continued research and clinical trials are essential to developing new treatments to improve patient outcomes significantly in this challenging area.

## Data Availability

No datasets were generated or analysed during the current study.
